# Revisiting the Obligation to Share Aggregate Results with Research Participants in the Era of Open Science

**DOI:** 10.1002/eahr.60009

**Published:** 2025-07-14

**Authors:** Katherine E. MacDuffie, Benjamin S. Wilfond, Stephanie A. Kraft

**Affiliations:** ^1^ Assistant professor at the Treuman Katz Center for Pediatric Bioethics and Palliative Care in the Seattle Children's Research Institute and Department of Pediatrics at the University of Washington School of Medicine; ^2^ Professor at the Treuman Katz Center for Pediatric Bioethics and Palliative Care in the Seattle Children's Research Institute and Department of Pediatrics at the University of Washington School of Medicine; ^3^ Assistant professor in the Department of Bioethics and Decision Sciences at the Geisinger College of Health Sciences

**Keywords:** open science, open access, data sharing, data repositories, aggregate research results, research participants, human subjects research, human research ethics

## Abstract

Open science initiatives, intended to democratize access to research products, have made steady progress in shifting the global science culture toward practices like preregistration and data sharing. However, current open science efforts have not yet addressed the special obligation to ensure that research results are made accessible to the portion of the general population who contribute most directly to scientific advances: research participants. In this article, we explore the ethical obligation to communicate aggregate results to research participants and consider elements of open science infrastructure that could be amended for this purpose. We consider open questions for implementation related to the methods, timing, potential harms, oversight, and incentives for communicating aggregate results and pose solutions that could, following the example of open science initiatives, succeed in nudging investigators to reciprocate the efforts of research participants by sharing the scientific findings they helped to advance.

Open science refers to practices, infrastructure, and policies intended to make the products of research—e.g., data and publications—available to all. The White House Office of Science and Technology Policy deemed 2023 the Year of Open Science,^1^ launching efforts like the National Institutes of Health's (NIH's) new policy on Data Management and Sharing (DMS), which requires that all grant submissions include a plan (and budget) for depositing resulting data into repositories that can be accessed free of charge, and initiating a requirement that by 2025 all peer‐reviewed publications resulting from federally funded research be immediately publicly available. The ethical rationale for these and other open science efforts is strong. Open science practices promote good data stewardship and scientific integrity, accelerate collaboration and discovery, and enable more efficient use of federal funding resources.^2^ At the broadest level, open science initiatives seek to change the culture of science such that practices like pre‐registration and data sharing are incorporated into all research projects from their inception.^3^

## OPEN TO WHOM?

Current open science efforts are focused on promoting “equitable” access to the products of research—deidentified datasets, for example, or peer‐reviewed publications.^4^ However, the “openness” as currently conceived is most likely to benefit *other scientists*, rather than the public per se.^5^ Open access does not mean that research products are truly *accessible*, as many members of society are not equipped with knowledge of how to access these products.

**Figure 1:**
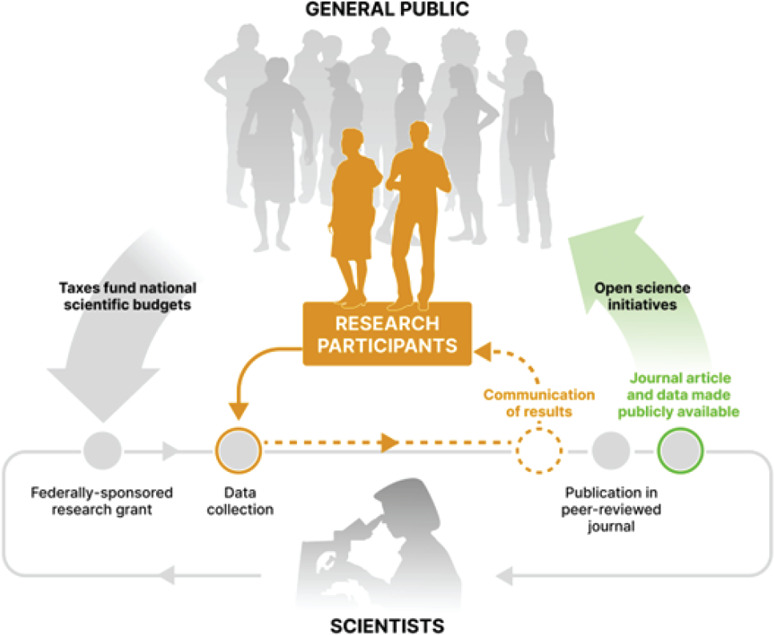
Incomplete Feedback Loop to Research Participants Open science initiatives aim to close the loop with the general public but do not address the direct contributions of research participants. Recent open science initiatives contribute to the cycle of scientific progress and make the products of science available to the general public, closing the feedback loop for research projects that are funded with taxpayer dollars. However, there are no guidelines or standard practices for sharing aggregate results with research participants—the portion of the public who contribute most directly to research—leaving this feedback loop incomplete.

Making research products accessible to those without advanced training and expertise is a worthy goal. However, we argue that current open science efforts should evolve to specifically acknowledge the special contributions of the portion of the public who contribute most directly to research—research participants (figure 1). Because of this contributory role, sharing the products of research with research participants deserves dedicated attention beyond the overall need to improve public access to the products of science.

## THE OBLIGATION TO SHARE AGGREGATE RESULTS DIRECTLY WITH RESEARCH PARTICIPANTS

Learning the results of the research in which one participates—and obtaining results that are understandable and in an accessible format—is a demonstration of respect and a bare‐minimum fulfillment of what is owed to research participants.^6^ The Declaration of Helsinki and other benchmarks for ethical human subjects research adopt the position that researchers should share results with research participants.^7^ Sharing research results guards against treating participants as mere objects of study but instead acknowledges them as fellow human beings—thoughtful, inquisitive, and curious—for whom the experience of participating in research may be deeply meaningful.^8^

Decades of research have documented a near‐universal desire of participants to learn the results of research they participate in.^9^ This is not surprising given that the research participants typically report a desire to help others—often described as an altruistic tendency—as a top motivation for participating.^10^ People participate in research because they want to help, yet rarely receive feedback about how they have helped. While it is sometimes feasible to share individual health‐related results with identifiable participants, the increasing practice of secondary research with large, deidentified datasets often renders individual result communication impossible. However, aggregate study findings can be made available to participants even when communication of individual results is not feasible or appropriate.^11^

Communicating aggregate results can fulfill the altruistic expectations that motivate participation in research in the first place, and potentially even encourage participation. Motivation to help others is stronger when people can expect to receive feedback about how their actions actually helped.^12^ This potential to improve recruitment and retention is a central advantage of communicating aggregate results, but not the only potential benefit for research teams.^13^ Dresser has written on the value of having nonscientist members on review committees as providing “a constant reminder of the outside world,” ensuring that research activities “must be defensible to the broader society, whose support is necessary to continue these activities.”^14^ Similarly, sharing results with research participants could serve as a reminder—one that is both motivating and humbling—to researchers of the underlying question or problem in the “outside world” that their research seeks to address and the need to pursue research questions that are meaningful to the broader society and, most specifically, to the people they ask to participate.

In line with these arguments, investigators report feeling an ethical obligation to share results with research participants.^15^ And yet, a survey of authors of PubMed‐indexed trials found that only 27% had already shared results with participants and 13% intended to do so, while 33% had no intentions to share and the remaining 27% were unsure or did not indicate their dissemination plans.^16^ Investigators cite a number of barriers to following through on the intention to share results, including lack of dedicated resources following the conclusion of a project, lack of incentives when investigators are primarily rewarded for publishing peer reviewed manuscripts, institutional review board policies that hinder ongoing communication with participants, and uncertainty about how to share results.^17^ These data suggest that communicating results to research participants has, to date, mostly relied on the personal efforts and the good will of researchers, operating without guidance or support from their funders or institutions.^18^ Institutional resources should be invested to help investigators meet this ethical obligation, and to prioritize it among the many other obligations that compete for time and attention.

## BUILDING ON OPEN SCIENCE INFRASTRUCTURE

Open science initiatives in the U.S. and abroad have made clear that establishing supportive tools and infrastructure are necessary to shift research norms and practice.^19^ Lessons learned from these processes, and even some of the tools themselves, could be modified to include aggregate results communication to research participants.

The NIH's DMS plans, for example, could be broadened to include results communication. As of January 25, 2023, all NIH research grant applications

**Communicating aggregate results can fulfill the altruistic expectations that motivate participation in research in the first place, and potentially even encourage participation**.

must include a DMS plan which outlines a process for curating and sharing (i.e., via open access repository) all resulting data. NIH institutes and centers have created templates and model DMS plans for a broad range of project types. Perhaps most importantly, investigators are required to include a section for DMS‐associated costs in grant budgets and are similarly provided with resources to guide the budgeting process. DMS plans are not reviewed by peer reviewers, but rather are assessed by program staff and can be modified over the course of a project if needed.

The structure of the NIH's DMS plan is ideally suited for incorporating plans to communicate aggregate results to participants and could overcome many of the commonly cited barriers to following through on the intention to share results. When a communication plan is not included from the beginning of a project, investigators tend to face barriers such as needing to modify an existing IRB protocol to allow for additional contact with participants, or having no budgetary resources or staff time dedicated to communication.^20^ If a plan for communication of research results to participants were incorporated into DMS plans at the proposal stage, investigators would benefit from a central source of templates and examples, and from proactively incorporating communication plans into IRB protocols and budgeting for communication efforts prior to launching their research.

Plain language summaries are another open science initiative that could facilitate results communication to research participants.^21^ Academic publications are not written in language that is accessible to most members of the general population (and, by extension, most research participants). In recognition of this, investigators are increasingly asked to write “plain language summaries” of research findings that can convey results in simpler language than typical in academic writing.^22^ As of 2022, the European Union Clinical Trials Regulation requires that investigators publish plain language summaries six months after study completion,^23^ and funders like the NIH and the National Science Foundation similarly require a plain language summary of grant results to be included as part of final progress reports, which are posted publicly on funder websites. Given that investigators are already creating plain language summaries of research results in all of these cases—perhaps with the assistance of large language models like ChatGPT that are adept at summarizing complex biomedical content^24^—sharing these summaries directly with study participants could be relatively straightforward. Indeed, one funder in the U.S., Patient‐Centered Outcomes Research Institute (PCORI), has already created infrastructure for sharing study summaries with participants. At the conclusion of a study, PCORI staff create plain language summaries of findings for investigator teams, which are posted on the PCORI website after approval. PCORI applicants are asked to budget up to $2500 in their initial grant proposals and to make “reasonable efforts” to share these plain language summaries with study participants.^25^

## OPEN QUESTIONS FOR IMPLEMENTATION

A number of important questions remain about how to share results with research participants that require careful thought and additional scholarship.

### What are evidence‐based methods for sharing results, and what are desired outcomes?

A limited amount of existing research has directly tested methods for communicating aggregate results to research participants. A recent scoping review^26^ described only two studies that used randomization to experimentally evaluate the outcomes of sharing aggregate clinical trial results. Participants in a breast cancer trial randomized to receive trial results via an online summary had a better understanding of results compared to control group participants who received results in other ways (e.g., by speaking with their oncologist.)^27^ Participants in a hypothyroidism trial randomized to receive trial results in a patient‐based letter format did not demonstrate improved understanding compared to those randomized to receive a press release describing study findings.^28^

Improving participant understanding of trial results is a plausible outcome, but what are the broader goals of sharing aggregate results? What should investigators hope to achieve by communicating results with participants, beyond an ethical obligation to demonstrate respect? For longitudinal research, participant retention is an important goal. Improving participant satisfaction with the research experience in and of itself may have less short‐term instrumental value than retention, but if higher satisfaction yielded greater willingness to participate in future research, that would be of great benefit to the broader research field. Measuring and tracking impacts of results communication on understanding, satisfaction, trust, and future research participation poses several challenges, but could be possible in certain settings where researchers and structured communities are mutually engaged over time.

### When should aggregate results be communicated?

Existing models for results communication have focused on data sharing at the end of a study, often at the time of publication. This is the same timing enforced for DMS plans, where data must be deposited at the end of a grant's performance period or at the time of an associated publication, whichever comes first. There are good reasons for waiting until one of these two time periods to communicate results. However, the pace of science is slow, and there could be value in sharing interim results with participants (even as simple as: “enrollment is ongoing, we are 2/3 of the way there,” or “enrollment is closed and data analysis has begun”). Interim updates of the sort could be given annually, encouraged, or required as part of grant progress reports or continuing reviews for the IRB. While it may not be appropriate in all cases, sharing such interim progress updates could serve as an accountability check for researchers (as previously quoted, “a constant reminder of the outside world”^29^) and also serve an educational purpose, showing participants first‐hand how the process of science can be slow, that it is influenced by global events like pandemics and national budget cuts, and often does not generate results with immediate splashy significance. This type of transparency could, if implemented broadly, improve broader public understanding of the scientific process, and perhaps contribute to rebuilding public trust in science, which is currently at historic lows.^30^

### What about potential harms of aggregate results?

One source of hesitation to communicate aggregate results is a concern that they could cause harm. Participants could become discouraged by negative or null results, they could overinterpret results that are not intended for clinical use, or, for some particularly small or potentially identifiable communities, results could create community harms in the form of increased stigma or countering identity narratives.^31^ These concerns in some cases are warranted. However, in many cases they are overly paternalistic. In the era of open science, the notion that researchers could cause more harm by directly communicating aggregate results to participants compared to a participant “discovering” an open‐access publication on their own is hard to defend.^32^

When there is potential that sharing aggregate results could result in either individual or community harm, research participants and community members can provide guidance. There are good examples in the literature of how community engagement has shaped communication of results for the better.^33^ Indeed, consultation with a participant or community advisory board about communication of aggregate results would probably be beneficial in all cases but is particularly necessary for research on topics that are sensitive or that involve identifiable groups. Individual participants could also be given the option to receive aggregate results at the conclusion of the study or not. An opt‐out option on a consent form could easily capture such preferences and assuage investigator concerns about unwelcome communication.^34^ Routinely collecting such preference data could also demonstrate to researchers the degree to which participants desire to learn aggregate results, even in quite sensitive research contexts.

### Should aggregate results sharing be required, or required for only some types of human subjects research?

The U.K. and France now require communication of aggregate clinical trial results to participants.^35^ In the U.S., research funders like the NIH^36^ strongly encourage sharing results with clinical trial participants, but do not yet require it.

The specific focus of these recommendations/requirements on clinical trials is perhaps not surprising, given the additional regulations to which such studies are already subject. However, it is unclear how the ethical obligation to share aggregate results with research participants in clinical trials is different from other human subjects research.^37^ As described above, longitudinal research, much of which is purely observational, may be a setting in which communicating results is particularly impactful for motivating retention over time. Indeed, the ethical obligation may be strongest for studies that offer no prospect of direct benefit. If the only promised benefit of a study is to advance scientific knowledge, sharing with participants how their contribution advanced knowledge holds particular salience. Put another way: the stronger the appeal to altruism when recruiting participants, the stronger too may be the obligation to share aggregate results.

The question of whether to require or recommend is challenging. The current “strongly encourage” language employed by the NIH and others does not seem to be having the intended effect of changing investigator behavior,^38^ suggesting a requirement might have more force. On the other hand, a requirement could create resentment and result in researchers doing the bare minimum to “check the box,” and in the process, largely miss the intent of creating accessible communication materials.

Following the model of the NIH's DMS plan could again be helpful here. Submitting a DMS plan with NIH grant applications is now required, but the data sharing itself is not. The act of requiring a plan means that researchers must think about what data they will generate, how they might share it (or why not), and with whom. A requirement as part of a funding application that researchers address whether and how they will communicate aggregate results to research participants could go a long way toward increasing awareness of the practice and ultimately nudging researchers toward communicating results without creating resentment or compliance fatigue. Similarly, IRB protocol templates could require that researchers discuss their (optional) plans for aggregate results communication prior to launching a study, thus encouraging investigators to at least consider results communication, and to set themselves up for success if they decide to share results midway through or at the conclusion of the study.

### Who should incentivize and support results communication?

We have placed considerable emphasis on the potential role of funders for encouraging results communication, given the leading role they play in promoting open science practices and shaping the culture of research.^39^ IRBs could also play a role here, but it is likely that many IRBs may consider oversight of aggregate results sharing activities as outside of scope given their focus on ensuring compliance with human subjects regulations, which in the U.S. do not include aggregate results sharing.

Academic institutions, in contrast, are well positioned to play a role in incentivizing results communication by their investigators. Open science initiatives have encouraged institutions to consider practices like preregistration, replication, and data sharing in tenure and promotion decisions,^40^ and uptake of such standards by academic departments has been publicized.^41^ Sharing results with research participants could be folded into these efforts to incentivize open science and be similarly encouraged and rewarded by academic institutions.

Finally, institutions could support results communication by providing infrastructure to make it easier for investigators to follow through with an intention to share results. For example, academic departments or research centers could dedicate web team resources to helping investigators host public‐facing summaries of research results on departmental websites. Developing a local culture of results communication within a given department or institution—while providing supportive infrastructure for investigators who have good intentions but limited time—could create an environment in which communicating aggregate results to research participants is sustainably prioritized.

## CONCLUSION

In the era of open science, researchers should proactively share results with the portion of the public who contribute most directly to research by giving their time, energy, and access to their thoughts, feelings, bodies, or data. Components of the open science infrastructure are particularly amenable to incorporating plans for communicating aggregate results to participants, and while some challenges remain for implementation, none are insurmountable. By sharing aggregate results, researchers can respect and reciprocate the altruistic urges that motivate research participation in the first place and in so doing, perhaps even encourage more people to serve as participants in biomedical research.

## ACKNOWLEDGMENTS

Many thanks to our colleagues in the Treuman Katz Center, as well as Malia Fullerton and Meg Doerr, for their feedback on earlier drafts.
